# A new patient safety smartphone application for prevention of “forgotten” ureteral stents: results from a clinical pilot study in 194 patients

**DOI:** 10.1186/s13037-017-0123-3

**Published:** 2017-04-04

**Authors:** Wilson R. Molina, Rodrigo Pessoa, Rodrigo Donalisio da Silva, McCabe C. Kenny, Diedra Gustafson, Leticia Nogueira, Mark E. Leo, Michael K. Yu, Fernando J. Kim

**Affiliations:** 1 Denver Health Medical Center, Denver, USA; 2 University of Colorado Denver, Denver, USA; 3 Urology Specialists of Nevada, Las Vegas, USA; 4 Dayton Physicians, LLC, Ohio, USA

**Keywords:** Stents, Renal Stone, Ureteroscopy, Urolithiasis, Patient safety

## Abstract

**Background:**

Approximately 12% of all ureteral stents placed are retained or “forgotten.” Forgotten stents are associated with significant safety concerns as well as increased costs and legal issues. Retained ureteral stents (RUS) often occur due to lack of clinical follow-up, communication or language barriers, and economic concerns.

**Methods:**

We describe a multiplatform application that facilitates data collection to prevent RUS. The “Stent Tracker” application can be installed on mobile devices and computers. The encrypted and password-protected information is accessible from any device and provides information about each procedure, stent placement and removal dates, as well as product description. This multicenter retrospective study included 194 patients who underwent stent placement between July and October 2015. Nominal data was tallied and ordinal data was divided into quartiles of 25, 50, and 75%.

**Results:**

A total of 194 patients from three institutions underwent ureteral stent placement. Reasons for stent placement include 122 cases post ureteroscopy (63%), 8 cases post percutaneous nephrolithotomy (PCNL) (4%), 14 cases post extracorporeal shock wave lithotripsy (SWL) (7%), 18 cases of cancer-related ureteral obstruction (9%), 21 cases of hydronephrosis (11%), and 11 for other reasons (6%). Of these patients, only one patient was lost to follow-up (0.5%). On average, ureteral stents were removed within 14 days of placement (IQR: 8-26 days).

**Conclusions:**

The “Stent Tracker” is a patient safety application that provides a secure and simplified interface, which can significantly reduce the incidence of RUS. Further developments could include automated notifications to patients and staff, color-coding, and integrated information with electronic patient charts.

## Background

Ureteral stents are an integral part of the treatment of different urological conditions such as nephrolithiasis, ureteral stricture, malignant obstruction, ureteral injury and healing [1, 2]. Retained ureteral stents (RUS) may cause infection, encrustation, and patient discomfort. Therefore, the use of ureteral stents require timely follow-up in order to avoid increased morbidity, mortality, and healthcare costs [3].

el-Faqih et al. reported that, following stents placed for urolithiasis, encrustation occurred in 9.2% of those removed under 6 weeks, 47.5% of those removed between six and 12 weeks, and 76.3% of those removed after 12 weeks [2]. Treating encrusted stents usually requires multiple procedures, significantly affecting costs. Sancaktutar et al. demonstrated that the cost of treatment for RUS was higher than a timely stent extraction and the financial burden of the treatments increased at the same rate as the duration of the stent retention (*p* = 0.001) [4].

Stent tracking systems based on patient registries have been used to reduce the incidence of RUS. Card-based versions of stent tracking, as described by Tang et al., have been advocated in the past but were largely replaced by electronic registries with automated reminders that link stent information to the patient’s electronic medical record [3, 5–8]. Ather et al. reported a substantial decrease in the incidence of forgotten stents from 12.5 to 1.5% over the course of 1 year, making a strong case for the implementation of such systems into urological practice [5]. We describe a patient safety software that facilitates data collection to prevent RUS. To our knowledge, this is the first report of a smartphone-based platform for stent tracking in clinical practice.

## Methods

**Fig. 1:**
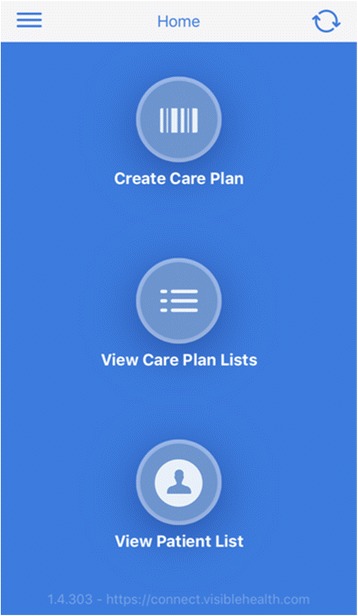
Snapshot of home screen of the “Stent Tracker” app

**Fig. 2:**
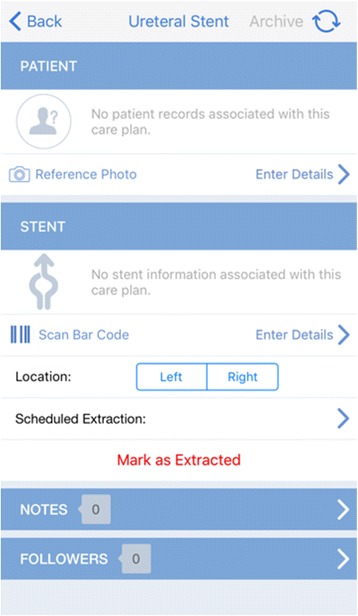
Snapshot of patient profile page on the “Stent Tracker” app

The new application “Stent Tracker” was developed by Visible Health in partnership with Boston Scientific [9]. It aims to improve patient safety, facilitate data collection, and provide an efficient interface to simplify ureteral stent tracking. The “Stent Tracker” application can be downloaded from the Apple store and installed on mobile devices and computers. However, only Boston Scientific stents can be tracked in this application and its use is limited to physicians who are pre-authorized and registered by Boston Scientific. The encrypted and password-protected application provides information about each procedure, scheduled removal dates, and product description for Boston Scientific ureteral stents [10, 11]. (Figures 1 and 2). The application encrypts all data, both in transit and at rest. Visible Health uses Amazon Web Services for database encryption [10]. Visible Health’s security officer, privacy officer, and application administrator have access to data for maintenance and user support. Visible Heath’s privacy policy is compliant with Health Insurance Portability and Accountability Act (HIPAA). The password protected software offers three options: create a care plan, view care plan lists, and view the patient list. Creating a care plan is simplified by scanning the ureteral stent’s barcode. The software allows the healthcare provider to review all patient data divided into sections of overdue cases, incomplete cases (missing information), indwelling stents, and extracted stents.

The data was acquired from three institutions and a retrospective analysis was performed which included patients who had stents placed between July and October 2015. Stents were considered overdue when they were left in place for longer than anticipated. Nominal data were tallied; ordinal data were divided into quartiles of 25, 50, and 75%. Excel was used for descriptive analysis and data collection.

## Results

An initial group of 194 patients from three institutions underwent ureteral stent placement and were subsequently tracked using the “Stent Tracker” application. The descriptive analysis on this introductory series is summarized in Table 1. Mean patient age was 55.9 (43–65) years. Indications for stent placement included 122 cases post ureteroscopy (63%), 8 cases post PCNL (4%), 14 cases post SWL (7%), 18 cases of ureteral cancer-related obstructions (9%), 21 cases of hydronephrosis (11%), and 11 for other reasons (6%). Overall, 149 stents were removed as planned (77%), 17 stents were overdue (9%), and 27 were scheduled to be removed by the time of this analysis (14%). The average dwelling time for the 149 removed stents was 14 days (IQR: 8–26 days). One patient was lost to follow-up because they were unable to be contacted and had no permanent address (0.5%).

**Table 1:** Patient demographics and ureteral stent data

	Median (IQR)/Totals (%)
Cases (n)	194
Age (years)	55.9 (43–65)
Duration stented (days)	14 (8–26)
Side (n)	
Left Right	88 (46%) 105 (64%)
Stent Removal (n)	
As planned Overdue Lost follow-up Plan in-progress	149 (77%) 17 (9%) 1 (0.5%) 27 (14%)
Reason for stent placement (n)	
Post ureteroscopy Post PCNL Post ESWL Cancer Hydronephrosis Other	122 (63%) 8 (4%) 14 (7%) 18 (9%) 21 (11%) 11 (6%)

## Discussion

Forgotten ureteral stents can result in severe encrustation which may require challenging interventional extraction methods [12]. Previous studies have suggested that 48% of stents may become encrusted within 3-months post placement, which emphasizes the need for close follow-up [13]. Multiple levels of patient safety should be implemented as it has been proven that education may not be sufficient to prevent RUS. Monga et al. reported that 10% of their patients with RUS will fail to show-up for scheduled stent removal despite being counseled by healthcare professionals [14].

In addition to the morbidity related to RUS, the increased financial burden is another significant problem. Sancaktutar et al. reviewed 27 medical files of patients with forgotten stents that were referred to their clinic between 2007 and 2010 [4]. The cost of treatment was estimated to be, on average, 6.9 times higher than a timely stent extraction due to the need for additional radiological studies, interventions, medical treatments, and extended hospital stay [4].

Over recent years, many centers have concentrated their attention on medico-legal litigation regarding forgotten ureteral stents [15]. Osman et al. published a summary of the collected data on urological litigation within the United Kingdom in 2011. The details of all successful claims pertaining to urology were retrieved between 1995 and 2009 from their national database. Forgotten ureteral stents accounted for the largest number of successful postoperative negligence claims: 23 claims in 14 years [15]. This overtly emphasizes the importance of thorough clinical assessment, record keeping, and follow-up with patients after stent placement. Ultimate responsibility for timely removal of ureteral stents has to be shared among the surgeon, health organizations, and the patient.

Investigators have reported different methods to reduce the incidence of RUS by using both manual and computerized systems [3, 5–7]. McCahy et al. introduced their electronic system in 1991 to follow–up patients, which resulted in a reduction in late stent removal from 3.6 to 1.1% [16]. Ather et al. also noticed a reduction in the rate of overdue stents from 12.5 to 1.2% using an electronic stent tracker [5]. However, these systems had few advantages over the previously paper-based charts because they required manual data input and review.

Lynch et al. have popularized their electronic stent registration system that creates a case in the patient’s electronic chart whenever stent placement is documented and sets a mandatory maximum stent life [6]. When this period expires, the computer automatically sends email notices to medical personnel until the stent is removed and the database is updated. The main problem they encountered before introducing barcode technology was a low rate of data entry in the electronic registry; only 61% of the stents inserted in patients were being accurately quantified [6].

Subsequently, Sancaktutar et al. described the initial results of a computer-based system that tracks ureteral stents and automatically sends a reminder through a short message service (SMS) to both the patients’ and the urologists’ mobile phones [7]. A total of 186 patients received stents over an 11-month period, but only 108 were included in the group that was recalled by the stent extraction reminder program. The remaining patients were not included in the new system. From the designated due date to the time of stent removal, the mean delay was 307 ± 118.6 days in the group not participating in the project and 14.6 ± 2.06 days in the patients being tracked (*p* <0.0001) [7].

The use of a new application to track ureteral stents placed in 194 patients from different centers is described with the intent to reduce RUS. Only one patient was lost to follow-up and 9% of the stents were overdue at the time of removal, which marks significant improvement compared to previous reports. Conversely, median stent duration was similar to the best results from other series: 14 days (8–26). The application offers a safe, convenient manner for creating and following a list of patients, allowing information to be accessed as needed. Finally, the “Stent Tracker” facilitates data entry and may improve patient accountability and education preventing the incidence of forgotten stents.

## Conclusion

The “Stent Tracker” application is a patient safety tool which provides an encrypted and simplified interface that can reduce the incidence of retained or “forgotten” stents. Ultimately, the goal would be the elimination of forgotten stents and the complications associated with them. Other potential features that may be included are automated alerts and notifications to patients and staff, color-coding, and integrated information with electronic patient charts. Further developments of universal patient tracking platforms should contribute to a culture of safety for Endourological procedures.

## Abbreviations

IQR: Interquartile range
PCNL: Pecutaneous nephrolithotomy
RUS: Retained ureteral stents
SWL: Extracorporeal shock wave lithotripsy
